# Bacterial diversity among four healthcare-associated institutes in Taiwan

**DOI:** 10.1038/s41598-017-08679-3

**Published:** 2017-08-15

**Authors:** Chang-Hua Chen, Yaw-Ling Lin, Kuan-Hsueh Chen, Wen-Pei Chen, Zhao-Feng Chen, Han-Yueh Kuo, Hsueh-Fen Hung, Chuan Yi Tang, Ming-Li Liou

**Affiliations:** 1 Division of Infectious Diseases, Department of Internal Medicine, Changhua Christian Hospital, Changhua City, Taiwan; 2 Center of Infection Prevention and Control, Changhua Christian Hospital, Changhua City, Taiwan; 3 Department of Nursing, College of Medicine & Nursing, Hung Kuang University, Taichung City, Taiwan; 4 Department of Computer Science and Information Engineering, Providence University, Taichung City, Taiwan; 5 Department of Applied Chemistry, Providence University, Taichung City, Taiwan; 6 Department of Nursing, Yuanpei University of Medical Technology, Hsin-Chu City, Taiwan; 7 Department of Medicine, National Taiwan University Hospital Hsin-Chu Branch, Hsin-Chu City, Taiwan; 8 Department of Environmental Engineering and Health, Yuanpei University of Medical Technology, Hsin-Chu City, Taiwan; 9 Department of Medical Laboratory Science and Biotechnology, Yuanpei University of Medical Technology, Hsin-Chu City, Taiwan

## Abstract

Indoor microbial communities have important implications for human health, especially in health-care institutes (HCIs). The factors that determine the diversity and composition of microbiomes in a built environment remain unclear. Herein, we used 16S rRNA amplicon sequencing to investigate the relationships between building attributes and surface bacterial communities among four HCIs located in three buildings. We examined the surface bacterial communities and environmental parameters in the buildings supplied with different ventilation types and compared the results using a Dirichlet multinomial mixture (DMM)-based approach. A total of 203 samples from the four HCIs were analyzed. Four bacterial communities were grouped using the DMM-based approach, which were highly similar to those in the 4 HCIs. The α-diversity and β-diversity in the naturally ventilated building were different from the conditioner-ventilated building. The bacterial source composition varied across each building. Nine genera were found as the core microbiota shared by all the areas, of which *Acinetobacter*, *Enterobacter*, *Pseudomonas*, and *Staphylococcus* are regarded as healthcare-associated pathogens (HAPs). The observed relationship between environmental parameters such as core microbiota and surface bacterial diversity suggests that we might manage indoor environments by creating new sanitation protocols, adjusting the ventilation design, and further understanding the transmission routes of HAPs.

## Introduction

Humans live in health-care institutes (HCIs) during periods of hospitalization. The environments of HCIs are complex ecosystems that include trillions of microorganisms interacting with each other and with humans and their environment^1^. The ecology and diversity of the environmental microbiome in HCIs have important implications for patient health. Hospital environments are often overlooked reservoirs for these bacteria. The transmission of health-care-associated pathogens (HAPs) via surface contamination is now a critical issue in hospital-acquired infections (HAIs)^2^. Currently, HAIs remain a major cause of patient morbidity and mortality^3^. Most studies have focused on measures of the abundance of culturable or pathogenic strains, such as ‘ESKAPE’ organisms (*Enterococcus* spp., *Staphylococcus* spp., *Klebsiella* spp., *Acinetobacter* spp., *Pseudomonas aeruginosa*, and *Enterobacter spp.*), that most frequently cause HAIs^4^. However, a more comprehensive measure of diversity and the factors that determine the diversity and composition of the environmental microbiomes in HCIs are poorly understood.

Recently, the development of culture-independent, high–throughput molecular sequencing approaches has transformed the study of microbial diversity in different hospital locations, as demonstrated by the recent explosion of research in the microbial ecology of neonatal intensive care units (ICUs)^5^, respiratory care centers^6^, and medical ICUs^2, 7^. This molecular approach for exploring microbial diversity is based on the PCR amplification and sequencing of genes encoding small subunit ribosomal RNA (16S rRNA) directly from environmental sources^8^. This approach readily determines several orders of magnitude more microbial diversity than culture-based methods and has dramatically altered our understanding of microbial diversity. Using the 16S rRNA amplicon sequencing approaches, it has been concluded that the phylogenetic spectrum in hospital environments is closely aligned with potential human pathogens, implying that hospital environments are potential reservoirs for transmitting those pathogens. Even though the diversity and composition of the surface microbiome in an individual HCI has been determined, there have been few attempts to comprehensively survey the factors that determine the HCI microbiome.

Currently, the composition and diversity of microbiomes in a built environment are contributed to by two ecological processes: dispersal resources and the selection of certain microbial taxa by environmental conditions^9^. The dispersal resources likely come from outside air, indoor surfaces, and the bodies of humans and other micro- and macro-organisms residing and moving through indoor spaces^10^. The selection of specific microbial types by the environment occurs due to air temperature and relatively humidity^11^, the source of ventilation air and occupant density^12, 13^, cleaning^2^, and decontamination of indoor air^14^, which can influence the abundance of some pathogenic microbes indoors. Previous reports showed that filtration by mechanical ventilation reduces the dispersal of outdoor microbes^15^. However, microbial taxa not commonly found outdoors were found indoors^1^. Thus far, it remains unclear which of the sources is the key determinant or what environmental factors might determine the relative abundance of bacteria within and among HCIs.

Here, we used the 16S rRNA amplicon sequencing approach to survey the environmental microbiome of four HCIs, consisting of two regional hospitals and two long-term care facilities (LTCFs). The four HCIs are located in three buildings: one regional hospital and one LTCF are located in the same building, and the remaining two are located apart from each other. We chose HCIs based on the fact that we can sample across a range of design and environmental factors (including ventilation source, temperature, humidity and occupant density recognized by carbon dioxide concentration) and because the surface microbiomes of HCIs have implications for patient health. We focused on the microbial diversity across the four HCIs with various environmental factors. Recently, a novel approach based on the Dirichlet multinomial mixture (DMM) for the probabilistic modeling of microbial metagenomics data has been deployed to better interpret the ecological dynamics underlying taxon abundance of microbial metagenomics data. While many methods used to classify or cluster samples have ignored features, such as samples have different size, and the sparse taxonomic distribution, as communities are diverse and skewed to rare taxa. The DMM approach describes each community by a vector of taxa probabilities. These vectors are generated from one of a finite number of Dirichlet mixture components, and the mixture components cluster communities into distinct type and groups of samples with a similar composition. The model can also deduce the impact of a treatment and be used for classification^16^. In this study, we also applied a DMM-based approach to investigate the community types among the four HCIs and compared them with the individual community of each HCI. We addressed four questions. First, what is the composition of surface microbiota in the four HCIs? Second, how does building design, particularly ventilation source, influence the diversity and structure of the microbiome? Third, what is the core microbiome, particularly for human-associated microbes, across the four HCIs? Fourth, what is the microbial source in each HCI?

## Results

### Parameter measurement in four HCIs

Table 1 summarizes the environmental and infectious parameters in each HCI. The air temperature, relative humidity, and mean concentration of CO_2_ in HCIs (TH, NH, and NC) with a central conditioner are somewhat different from those of the window-ventilated HCI (SC). The higher CO_2_ concentration in TH indicates the high occupant density in this hospital. Owing to window ventilation, the air temperature and relative humidity in SC were dynamic based on changes in the outdoor air. The cleaning strategies, which might affect the surface microbiome of the buildings^2^, were different in acute-care hospitals (TH and NH) than LTCFs (NC and SC). Four HCIs underwent terminal disinfection when a patient was discharged or had his bed changed. In hospitals, additional daily cleaning was required to prevent cross-infection among, in particular, immuno-compromised patients.

**Table 1:** Summary of the parameters measured within the four healthcare-associated institutes.

Institute	Location	Ventilation type	CO_2_ (ppm)	Temperature (°C)	Relative humidity (%)	Health care–associated infections, rate (‰)^c^	Cleaning strategies	Bed capacity
			Mean [Min-Max]	Mean [Min-Max]	Mean [Min-Max]			
TH^a^	Urban area	Central conditioner	1132 [826–1526]	24 [22–29]	61 [54–70]	1.53	TC^d^, DC^e^	550
SC^b^	Rural area	Natural	553 [458–818]	27 [20–30]	71 [58–81]	NA	TC	49
NH^a^	Urban area	Central conditioner	769 [568–935]	23 [22–24]	56 [49–66]	3.13	TC, DC	99
NC^b^	Urban area	Central conditioner	849 [567–1538]	24 [21–26]	65 [56–75]	NA	TC	49

^a^Acute-care hospital; ^b^Long-term care facility; ^c^Per 1000 patient-days; ^d^Terminal cleaning; ^e^Daily cleaning.

Notes: The terminal disinfection and daily cleaning procedure followed the description of Chen *et al*.^2^.

### Sample collection and sequencing

A total of 203 samples from the four HCIs were collected between January and December 2015. These samples were classified into three groups, i.e., workplaces, high-touch areas, and environments, as shown in Table S1. Following DNA extraction and barcoded PCR amplification, a high number of amplicons were obtained and sequenced (Table 2). In total, the raw dataset of 203 samples contained 11,381,154 sequences. After trimming, the average number of OTUs per sample was 339 in NC and 933 in SC. An OTU is defined here as organisms sharing ≧97% 16S rRNA gene sequence identity. Due to the different numbers of sequences among samples, the data were normalized by the total sequence reads within a sample as relative abundance.

**Table 2:** Summary description of the sampling sites, the number of sequences collected, and the level of bacterial diversity identified.

	TH	SC	NH	NC
Total no. of sequence reads	11,381,154			
Average length of sequence reads before merging bp (range)	301 (301–301)			
Average length of sequence reads after merging bp (range)	336.92 (61–490)			
No. of sampling sites	116	35	28	24
Total no. of classifiable sequences	7,208,513	1,582,210	739,076	898,228
Total no. of OTUs in all samples	8,504	5,959	2,194	3,399
Average no. of sequences per sample (range)	62,142 (2,056–439,086)	45,206 (1,789–122,052)	26,395 (2,710–183,090)	37,426 (1,574–133,633)
Average no. of OTUs per sample (range)	796 (238–2,617)	933 (326–1,807)	543 (147–1,421)	339 (139–1,077)

Phylotypes were determined at the 97% sequence similarity level.

### Taxonomic composition of the surface microbiota

Figure 2 shows the relative abundance of bacterial genera in four HCIs. As shown in Figure 2A, the microbial communities included 8 different phyla: *Proteobacteria, Fusobacteria, Firmicutes, Deinococcus-thermus, Cyanobacteria, Candidate_Division_TM7, Bacteroidetes* and *Actinobacteria. Proteobacteria* (22%) was the most abundant phylum across all of the samples. At the genus level, only 9 genera, including *Pseudomonas, Massilia, Enterobacter, Acinetobacter, Staphylococcus, Chroococcidiopsis, Dysgonomonas, Propionibacterium* and *Corynebacterium*, were retrieved from surfaces of all four HCIs. The spectrums of microbial communities in the 3 HCIs with a central conditioner (TH, NH, and NC) were different than SC. *Dysgonomonas* (18–53%) was the most abundant genus in TH, NH, and NC, whereas *Staphylococcus* (19%) was the most abundant genus in SC.

**Figure 1:**
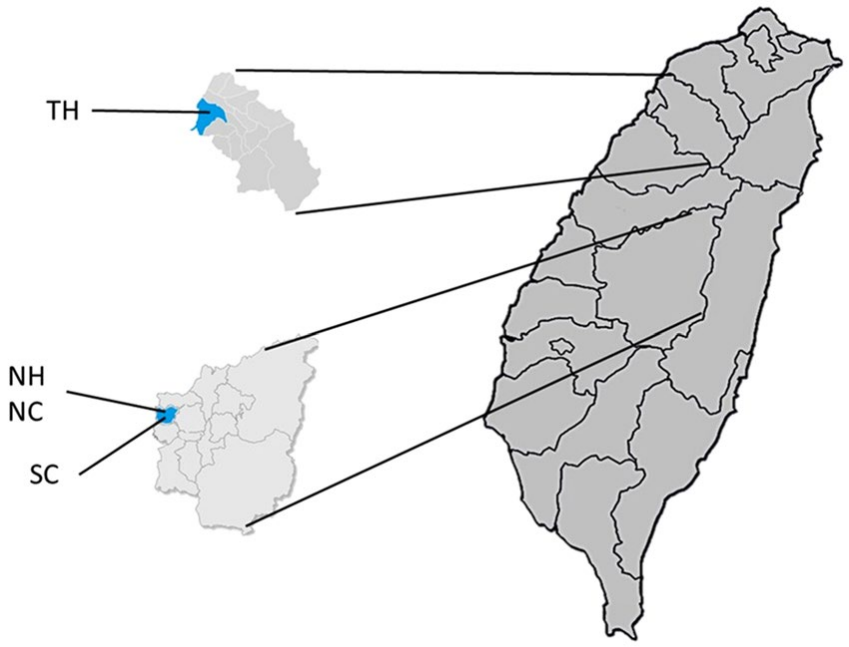
Sample collection map. Samples were collected from acute care hospitals (TH and NH) and long-term care facilities (NC and SC). NH and NC are located in the same building. Chen CH and Chen KH generate three maps using Ulead Photoimpact software version 12.0 (http://www.paintshoppro.com/tw/products/photoimpact/).

**Figure 2:**
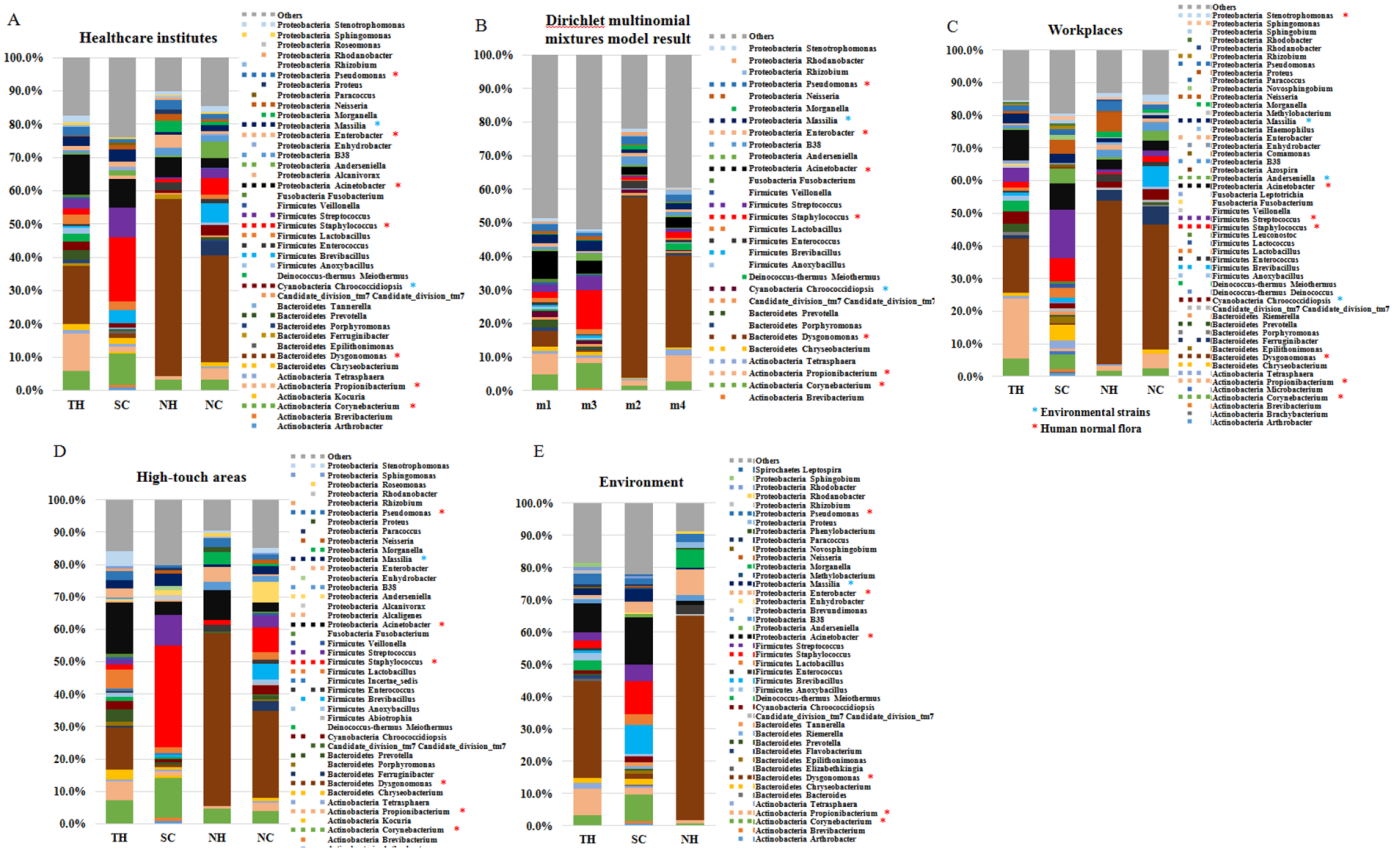
The bacterial communities of four healthcare-associated institutes (**A**). The bacterial communities from the sampling sites in each institute were classified by the DMM-based method (**B**). The bacterial communities from the sampling sites in each institute were classified into workplaces (**C**), high-touch areas (**D**), and environments (**E**). The relative composition of the genera was determined by 16S rRNA amplicon sequencing. Only each healthcare institutes’ genera with >0.5% abundance on average were included. Multi-colored charts in the legend are shown for each genus and sample.

To clarify the difference in bacterial communities among the four HCIs, we performed DMM-based community typing based on the genus-level envirotype observed in each of 203 samples. We identified four community types supported by the smallest Laplace value (Laplace = 258,753), as shown in Figure S1. Figure 2B represents the top 30 genera across the four community types. The four community types analyzed by DMM were highly similar to those among the four HCIs, as shown in Figure 2A. For example, one cluster (m2) was dominated by *Dysgonomonas* and was compatible with the NH envirotype, and one cluster (m3) was dominated by *Staphylococcus* and was compatible with the SC envirotype. The other two (m1 and m4) had a more variable community composition, and they were compatible with TH and NC, respectively.

A closer investigation of bacterial communities in different areas among the four HCIs is shown in Figure 2C–E. The spectrums of microbial communities located in workplaces, high-touch areas, and environments were somewhat different in individual HCIs. In TH, *Propionibacterium* (19%) and *Dysgonomonas* (30%) were significantly abundant in workplaces and environments, respectively (*p* < 0.001). In SC, *Staphylococcus* (31%) was significantly abundant in the high-touch areas (Table S2). Nevertheless, *Dysgonomonas* remained the most abundant genus across the three areas in NC (27–38%), NH (50–63%), and TH (13–30%).

### Diversity of bacterial community profiles

**Figure 3:**
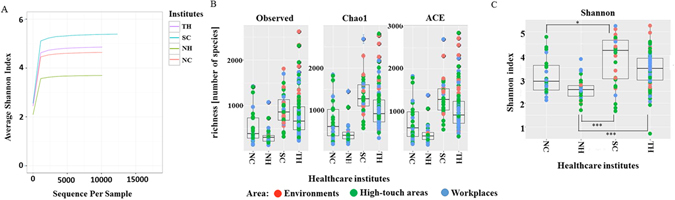
Alpha-diversity values with different sequencing depths were measured using Shannon index average scores (**A**). Alpha-diversity measurements for communities from the four healthcare institutes (TH, SC, NH, and NC). Alpha-diversity metrics were employed (richness and Shannon index) (**B**,**C**). The bottom and top of each box are the first and third quartiles, respectively, and the band inside the box is the median. The whiskers represent one standard deviation above and below the mean of the data. Statistical testing was based on the Wilcoxon rank-sum test (*p < 0.05, ***p < 0.001).

To determine the alpha diversity of the four HCIs, Shannon index curves scores and richness (Observed, Chao1, and Ace) were employed, as shown in Figure 3. Figure 3A shows that the Shannon diversity index curves clearly reached plateau levels after the sequence numbers exceeded 2,000 sequences in all four HCI’s, indicating that the bacterial genera composition for all four HCI was well-represented by these sequencing depths. Figure 3B shows the highest and the lowest measures of richness in SC and NH, respectively. The Shannon diversity index differed significantly between NH and SC (*p* < 0.001), NH and TH (*p* < 0.001), and NC and SC (*p* < 0.05) based on the Analysis of Variance (ANOVA) test and Scheffe post hoc test, as shown in Figure 3C. Table S3 shows alpha diversity in workplaces, high-touch areas, and environments in the four HCIs, and the areas with the highest diversity are the workplace and environment for SC.

**Figure 4:**
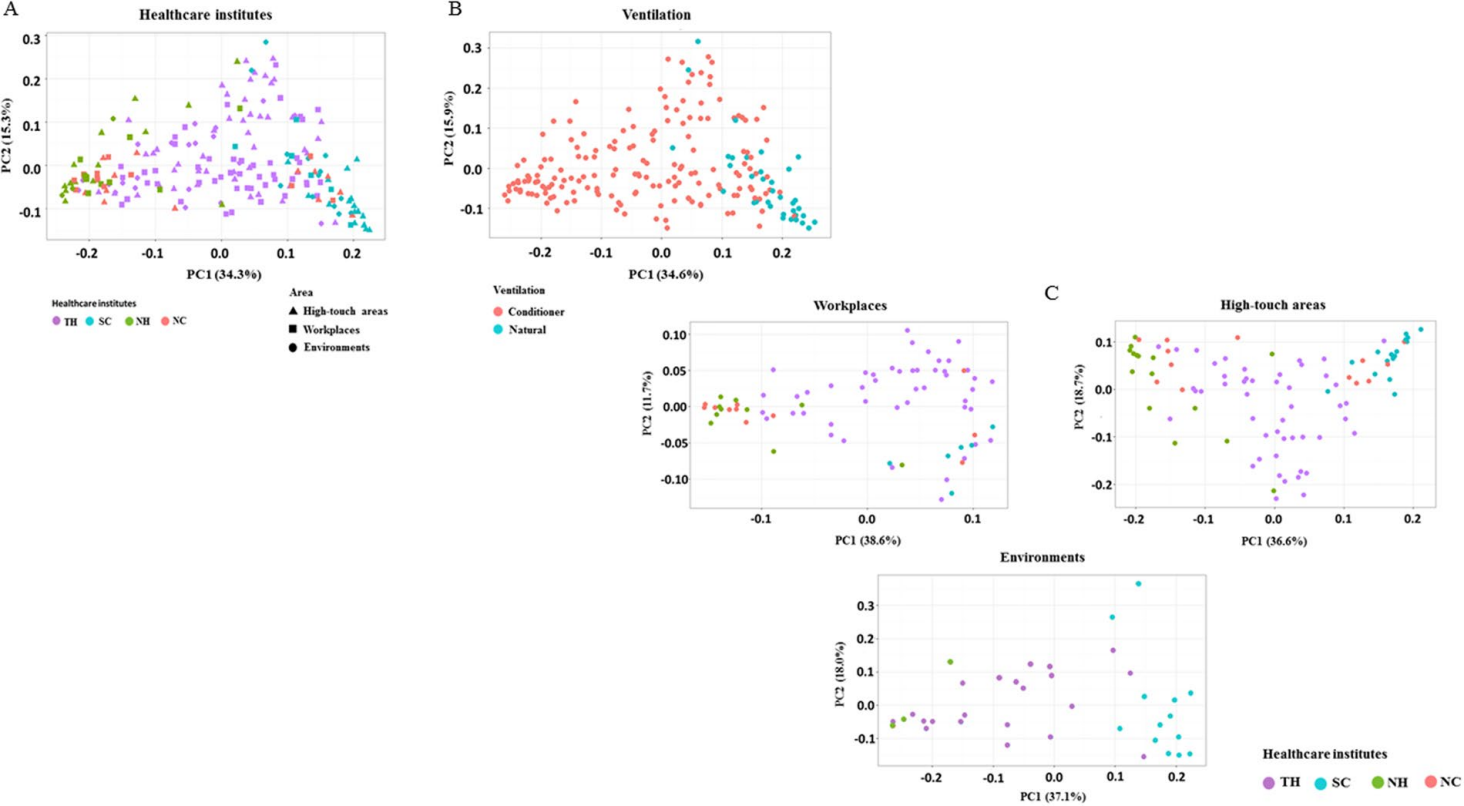
Bacterial communities associated with different areas among the four HCIs (**A**), different ventilation types (**B**), and workplaces, high-touch areas, and environments (**C**) by principal coordinate analysis (PCoA). PCoA plot based on the weighted UniFrac distance matrix. The percentage of diversity distribution explained by each axis is indicated in the figure.

To further explore the relationships among the different bacterial communities in the four HCIs, a PCoA analysis using a weighted Unifrac distance matrix was performed, and the result is shown in Figure 4. The beta-diversity of bacterial communities revealed an overlap between the bacterial populations among the four HCIs (Figure 4A). The SC and NH bacterial communities formed two distinct clusters from TH and NC. Some of the analyzed samples from NC cluster with NH and others cluster with SC. In addition, the analyzed samples from TH overlapped with the remaining three HCIs. Beta-diversity of bacterial communities with different ventilation types revealed different clusters between conditioner ventilated and naturally ventilated areas (Figure 4B). Additionally, bacterial communities revealed overlap among the three areas located in the three buildings (Figure 4C).

**Figure 5:**
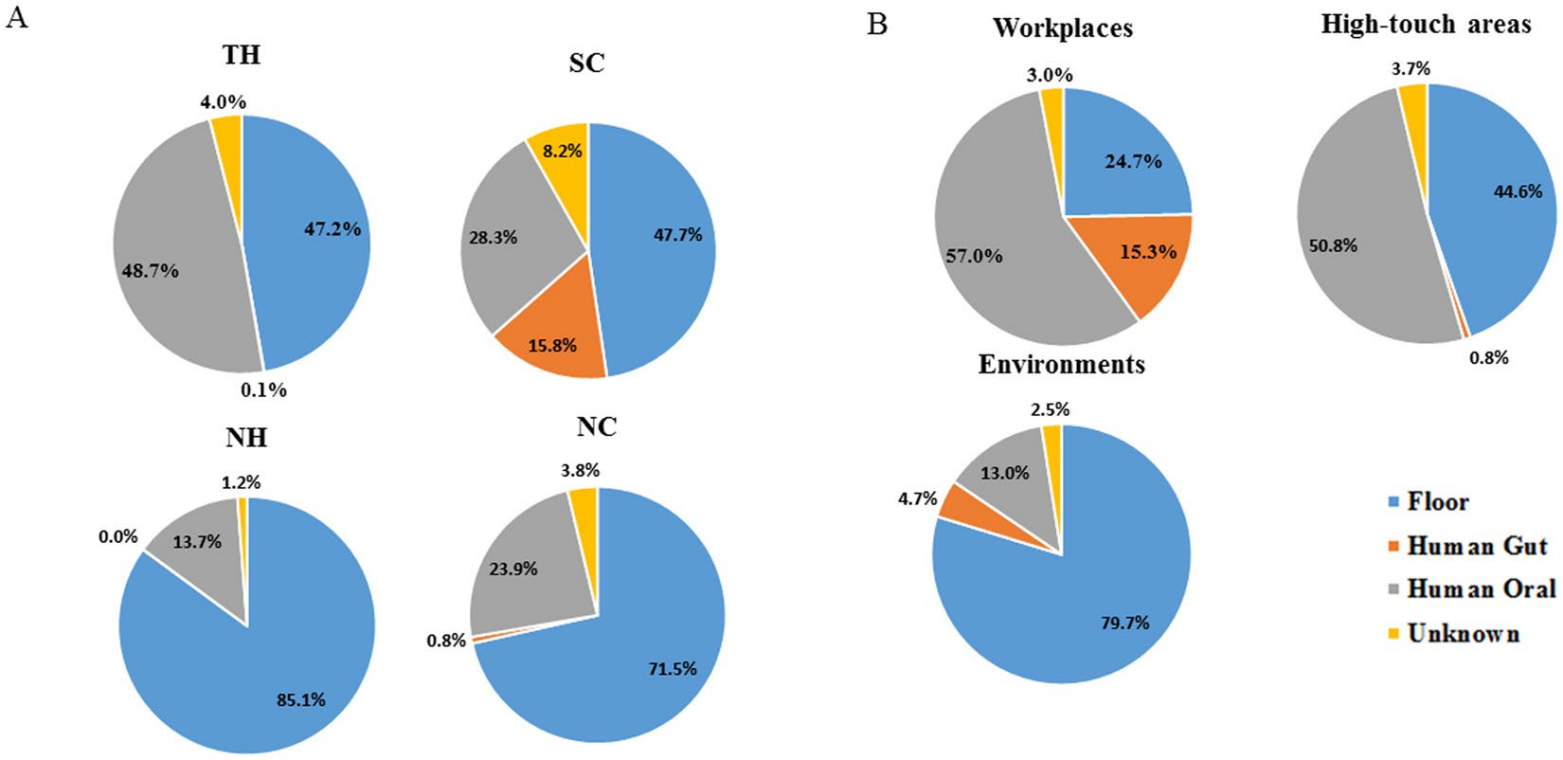
Source environment proportions were estimated using SourceTracker, and the results were shown in pie charts representing the mean proportions for the four healthcare-associated institutes (**A**) and three areas (**B**).

We used the SourceTracker software package to determine the potential sources of bacteria and how different sources varied across the environment surfaces of microbial communities in the four HCIs and the three environmental groups. The SourceTracker model assumes that each surface community is merely a mixture of communities deposited from other known or unknown source environments and, using a Bayesian approach, the model provides an estimate of the proportion of the surface community originating from each of the different sources. When a community contains a mixture of taxa that do not match any of the source environments, that portion of the community is assigned to an “unknown” source. Potential sources we examined included human mouth (n = 50), gut (feces) (n = 50), and floor communities (n = 11), whereas the environment in each HCI as sinks. The gut source samples were collected from patients and staffs of the four HCIs. The oral samples designed for the study of periodontitis were gathered from Taiwanese included 30 health persons and 20 patients with periodontal disease. The results illustrated in Figure 5 suggest that the source compositions in the same building (NH and NC) were highly similar but were different from the remaining two buildings (SC and TH). Further, the source compositions in high-touch areas and workplaces were closely related compared to environments.

### Core microbiome and human-associated microbes in four different HCIs

**Figure 6:**
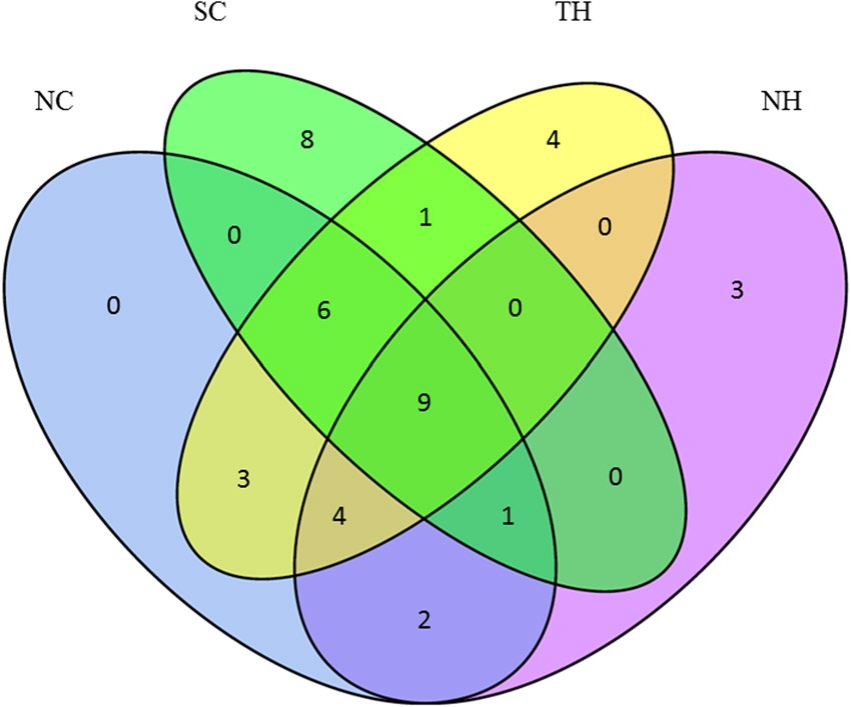
Venn diagram showing the number of shared genera between zones among the four HCIs.

A core taxon was defined as more than 0.5% mean relative abundance of the core genera shared by all the samples. The number of genera shared among different samples is represented in Figure 6. As shown in the Venn diagrams, 9 genera were shared between all samples of the four HCIs, which accounted for 58.03%. Although these genera were present in most samples, there was a significant difference in their abundance across groups (Table 3). For example, *Dysgonomonas* ranged between 53.13% (NH) and 1.15% (SC), whereas *Staphylococcus* ranged between 19.34% (SC) and 1.00% (NH). Among all of the core microbiota, seven genera belonged to human-associated microbes, including *Dysgonomonas, Acinetobacter, Staphylococcus, Pseudomonas, Corynebacterium, Propionibacterium* and *Enterobacter*. *Dysgonomonas* spp. (25.97%) remained the predominant genus, followed by *Acinetobacter* spp. (7.38%) and *Staphylococcus* spp. (6.77%).

**Table 3:** Mean relative abundances of the core genera that were shared by all the areas^a^.

Genera	TH	SC	NH	NC	Average
*Acinetobacter*	11.95	8.70	6.07	2.80	7.38
*Chroococcidiopsis*	2.77	1.33	0.86	3.08	2.01
*Corynebacterium*	5.80	9.42	3.19	3.25	5.41
*Dysgonomonas*	17.54	1.15	53.13	32.04	25.97
*Enterobacter*	1.44	1.46	3.78	0.80	1.87
*Massilia*	2.66	3.69	0.94	1.84	2.28
*Propionibacterium*	11.39	1.46	1.15	3.45	4.36
*Pseudomonas*	2.46	1.08	2.85	1.52	1.98
*Staphylococcus*	1.84	19.34	1.00	4.91	6.77

^a^The microbial species in this table are those identified as members of the core microbiota as reported in Figure 6.

### Comparison of five different healthcare-associated pathogens among four HCIs

The distribution of putative human-associated microbes, especially HAPs, remains the main focus of infection control in hospitals. Hence, we compared the HAPs among four HCIs. Table 4 shows the results of a Student’s t-test on the abundance of bacterial taxa in four HCIs. Comparing five different HAPs among different HCIs, (1) the abundance of *Acinetobacter* was significantly higher in TH compared to NH (*p* = 0.0307) and NH (*p* = 0.0348); (2) *Staphylococcus* had a significantly higher abundance in SC compared to the other areas; (3) *Streptococcus* was relatively more abundant in SC compared to the other areas; (4) *Pseudomonas* was more abundant in TH; and (5) *Enterococcus* had a significantly lower abundance in SC compared to the other areas.

**Table 4:** Results of Student’s t-test examining the bacterial cell abundances (%) in the four healthcare institutes.

	TH	SC	NH	NC	Student t-test					
	Mean (A)	Mean (B)	Mean (C)	Mean (D)	A/B	A/C	A/D	B/C	B/D	C/D
*Acinetobacter*	0.67	0.39	0.16	0.17	NS	0.0307	0.0348	NS	NS	NS
*Staphylococcus*	0.26	1.58	0.07	0.50	<0.0001	0.0028	NS	0.0001	0.0071	0.0289
*Streptococcus*	0.39	1.17	0.03	0.52	NS	0.0034	NS	0.0229	NS	NS
*Pseudomonas*	0.66	0.22	0.38	0.21	0.0005	NS	0.0001	NS	NS	NS
*Enterococcus*	0.53	0.09	0.86	0.44	<0.0001	NS	NS	0.0014	0.0096	NS

NS, not significant (*p* > 0.05).

## Discussion

Humans are the most important dispersal vectors for microorganisms indoors. The bacterial fingerprint of microorganisms represents a unique mix of bacteria around their environment^17^. In addition to the dispersal of microbes from humans or material surfaces, the diversity and distribution of microorganisms are also affected by variable environmental conditions, including temperature and relative humidity. For elderly or immunocompromised populations living in HCIs, the environmental microbiome has important implications for patient health, and HAIs remain a major concern^3^. In this study, we determined the diversity and composition of the environmental microbiome in HCIs and evaluated the relationships between significant HAPs among four HCIs. Herein, we have several findings. First, our results indicate that building design, particularly the source of ventilation air, does influence the diversity and composition of the microbiome of the built environment. Second, the bacterial fingerprints in the surface environment are restricted to the individual HCIs, even though the bacterial communities in the four HCIs overlapped. Third, human-associated microbiota, particularly HAPs, are predominant across all of the HCIs, implying that the potential risk for opportunistic infection might occur even though infection control strategies have been employed in those institutes. Fourth, multiple factors, including ventilation type, might contribute to the differences in diversity among the four HCIs based on our findings.

Our results indicate a much higher diversity of communities in the four HCIs using 16S rRNA amplicon sequencing^7^. The four dominant phyla were *Proteobacteria*, *Firmicutes*, *Actinobacteria*, and *Bacteriodetes*. These dominant phyla were also associated with previously built environment studies of public restroom surfaces^18^, gym surfaces^19^, and homes^20^. The same four phyla were also dominant on human skin^17^. Overall, the proportion of each detected phylum differed by 6–56% across the 4 HCIs, indicating that HCI community composition was highly variable, at least at the phylum level. Included in these phyla are sequences affiliated with *Fusobacteria, Cyanobacteria* and *Candidate_Division_TM7*. *Fusobacteria* was present on human skin^17^. *Cyanobacteria*, which has previously been found on classroom walls and floors, was attributed to soil and bioaerosol accumulation^21^. *Candidate_Division_TM7* is a highly ubiquitous phylum reported in soil, seawater, activated sludge and many animal- and human-associated sources^22^.

A closer investigation of genera distributed around the 4 HCIs revealed that there were two different community patterns between SC (window-ventilated) and the remaining 3 HCIs (air conditioner). A previous study has demonstrated that the observed variation in airborne microbial community structure in patient rooms at the sampled health-care facility was explained by the ventilation source^12^. The outdoor air communities were dominated by bacterial taxa common in aquatic and soil habitats^17^. Window-ventilated rooms contained potentially airborne bacterial communities intermediate in structure between mechanically ventilated patient rooms and outdoor air. In contrast, the indoor air communities were dominated by a small number of bacterial taxa that are commonly associated with humans as commensals or pathogens. Our previous study showed that up to 32% of *Staphylococcus* was present in the outdoor air of SC via the cultivation method (data not shown), indicating that the dominance of *Staphylococcus* in SC mainly came from outdoors. In this study, the observed variation in the potentially airborne microbial community profile among the four HCIs was explained by temperature and relative humidity, both of which are related to the ventilation source. Regarding ventilation with a central conditioner, we want to mention the role that air decontamination and increased airflow rate may play in reducing the contamination of environmental surfaces and the combined impact on interrupting the risk of pathogen spread^13, 14^. In contrast, the microbial community structure of an institutional surface would be merged into the natural environmental microbiome under the natural ventilation type. At least 3 species of bacteria (*S. aureus*, *K. pneumoniae*, and *P. aeruginosa*) are recommended to test for by the U.S. Environmental Protection Agency’s guidelines^23^. Some suitable work has been described^13, 14^, and the studies also comply with the U.S. Environmental Protection Agency’s guidelines^23^ for testing air decontamination technologies. It is critical to develop the recommended methods for air decontamination technologies in HCIs, including in Taiwan.

The composition and diversity of microbiomes of the built environment have been contributed to by dispersal resources and indoor environmental conditions. Considering three HCIs localized in two buildings with air conditioners, alpha-diversity and beta-diversity evaluations of the bacterial communities showed that NC and NH were highly similar, and they differed from TH. NC and NH were localized in the same building. Except for differences in cleaning strategies, environmental parameters such as temperature, relative humidity, and carbon dioxide concentration in the two institutes were quite similar. In contrast to NC and NH, a higher richness in alpha-diversity was observed in TH, which may be due to differences in ventilation efficiency and carbon dioxide concentration. Our findings reveal that, despite the building being ventilated with an air conditioner, the ventilation efficiency, including airflow rates and the filtration efficiency, and occupant density may play major roles in the diversity and distribution of microorganisms indoors. A limited number of HCIs is a weakness of our study, and it’s necessary to arrange further experiment for the impact of the natural ventilation condition.

In agreement with previous reports^24^, members of the core microbiota, such as *Dysgonomonas, Acinetobacter, Staphylococcus, Pseudomonas, Corynebacterium, Propionibacterium* and *Enterobacter*, can all be associated with human skin. The presence of skin-associated bacteria confirms that humans can be important dispersal vectors for microbes that colonize the built environment^25^. Whether *Dysgonomonas* sp. is a human pathogen remains controversial. Currently, six species of the genus *Dysgonomonas* with validly published names are recognized. These species were isolated from clinical sources, microbial fuel cells and the hindgut of a fungus-growing termite^26^.

Notably, *Acinetobacter*, *Staphylococcus*, *Pseudomonas*, and *Enterococcus* are regarded as HAPs. Previous studies have identified those dominant HAPs and their putative routes of transmission, such as physician and nursing staff clothing^27^, stethoscopes^28^, personal phones^29^, and computer keyboards^30^, using cultivation methods. In the last two decades, the widespread use and accumulation of antibiotics in the environment have caused a rapid increase in microbial resistance, which is largely driven by the transfer of anti-microbial-resistance genes. The high abundance of HAPs in the hospital environment implies that infection control strategies in those institutes would not be satisfactory, possibly due to the lack of manpower or the lack of proper execution. Those HAPs may be selected by antibiotics and then become high risk for patient health. Interestingly, our results show that *Enterococcus* was predominant in NC, NH, and TH. Based on clinical observations, one possible reason is that most of those three HCIs cared for critical and disabled patients, most of whom need their diapers changed, and hence *Enterococcus* was predominant in such hospital environments. In contrast, most of the residents in SC did not need diapers, and there was less *Enterococcus* in that environment. According to the analytical results of the SourceTracker, gut microbiota contributed 16% of the microbes in SC, followed by 0.4–0.8% in the remaining 3 HCIs. Thus, the spread of *Enterococcus* remains controversial. This study discovered that four types of HAPs, belonging to ‘ESKAPE’ organisms (*Enterococcus* spp., *Staphylococcus* spp., *Klebsiella* spp., *Acinetobacter* spp., *P. aeruginosa*, and *Enterobacter* spp.), are related to microbiomes of the built environment. Here, we describe a more comprehensive measure of diversity and composition of microbiomes of the built environment and hospital microbiomes in four HCIs. We want to mention their potential to influence hospital management policy and reduce HAIs.

## Conclusions

Our study discovered unexpectedly high bacterial community diversity, and hospital microbiomes were closely associated with microbiomes of the built environment. Although most hospital microbiomes can be considered non-pathogenic under normal circumstances, there are potential risks in the four HCIs where patients are extremely vulnerable to infections. Our study suggests that multiple factors, including ventilation type, might contribute to the differences in diversity among the four HCIs based on our findings. It is critical to develop the recommended methods for air decontamination technologies, and continuous monitoring of HAPs at HCIs would be required to reduce HAIs.

## Methods

### Ethical approval

This study was approved by Ethics Committee of the Changhua Christian Hospital (CCH IRB No. 140318) for collecting fecal samples as well as by Institutional Review Board of Chang Gung Memorial Hospital (Approval no. 102–4239B) for collecting oral samples. Each participant provided written informed consent under a protocol that was approved by the Institutional Review Board and all methods were carried out in accordance with these guidelines.

### Setting and study design

Four HCIs (including one LTCF named SC with 49 beds, another named NC with 49 beds, one acute-care hospital named NH with 99 ward beds of a community hospital in central Taiwan, and another acute-care hospital named TH with 550 ward beds of a regional teaching hospital located in northern Taiwan) were enrolled in this study between January and December 2015. Two HCIs, NC and NH, are localized in the same building. Three HCIs, TH, NH and NC, were mechanically ventilated, and SC was naturally ventilated (that is, primarily window-ventilated). Mechanically ventilated rooms had ventilation air supplied by the building’s heating, ventilation and air conditioning (HVAC) system through a supply duct and removed through a return duct and bathroom exhaust. Window-ventilation rooms had ventilation air supplied directly from the outside through windows. The geographic relationships are listed in Figure 1.

### Environmental measurements

Environmental samples were collected two to four times for each HCI during the study period. During each sampling period, environmental conditions, including air temperature, relative humidity and CO_2_ concentration, were measured by direct-reading monitors. A non-dispersive infrared (NDIR) sensor (Model G100, Geotech, Denver, Colorado, USA) working via spectroscopy was used to detect CO_2_, and measurements were recorded per minute. The room temperature and relative humidity were recorded by a thermo-hydrometer (Model 5330, Wisewind, Taipei, Taiwan) before and after surface sampling of microbes.

### Microbial sampling

Samples were collected in four patient rooms per HCI. Sampling sites around a bed in each HCI were chosen based on the frequency with which the surfaces were highly touched, and the sampling sites were divided into three groups: (1) workplaces, including keyboards, computer mice, curtains, mattresses, and quilts; (2) high-touch areas, including beds, monitors, ventilators, stethoscopes, oxygen supply, suction buttons, hemodialysis machines, intravenous pumps and feeding pumps; and (3) environments, including floors. The sampling sites collected from the four HCIs are listed in Table S1.

Microbes on the surface were sampled with sterile swabs. On the flat surfaces (i.e., ventilator screens and monitor screens), approximately 12 cm^2^ of each surface was swabbed. The computer mice were swabbed in their entirety, and a total of 10 keys on each keyboard were swabbed. After sampling, the protocols for DNA extraction and PCR were followed based on the description by Tang *et al*.^6^. Briefly, DNA was extracted directly using the MasterPure™ Gram Positive DNA Purification Kit (Epicentre, Madison, WI, USA). Following extraction, DNA was quantified using a fluorometer (Qubit; Invitrogen, Carlsbad, CA). PCR reactions were performed in small lots (two positive and one negative extraction, and two PCR controls) to reduce the possibility of laboratory contamination. Barcoded PCR amplification was performed using the V1 forward primer (5′-AGAGTTTGATCCTGGCTCAG-3′) and the V2 reverse primers (5′-TGCTGCCTCCCGTAGGAGT-3′) with 349-bp amplicons spanning the highly variable V1-V2 region of the 16S rRNA gene sequence of *E. coli* str. K12 substr. DH10B^31^. The PCR amplification was performed using 5X PCR Dye Master Mix II (GeneMark, Taichung, Taiwan). Each standard PCR volume contained 1 µl DNA sample, 500 nM of each primer, 4 µl 5X PCR Dye Master Mix solution and ddH_2_O up to 20 µl. The PCR was run with an initial 5 min denaturation at 94 °C, 30 cycles of 30 sec at 94 °C, 20 sec at 60 °C, 30 sec at 72 °C, and a final 5 min extension at 72 °C. Individual barcoded PCR products were purified and then pooled with a total combined concentration of 1 µg (total volume: 50 µl). All of the barcoded PCR fragments were sequenced using an Illumina Miseq Desktop Sequencer at Tri-I Biotech Inc. (Taipei, Taiwan). The raw sequences were deposited at the NCBI Sequence Read Archive under the Bioproject accession number PRJNA352047.

### Sequence processing

Paired-end reads sequenced by Illumina Sequencer were assembled with PEAR software^32^ (http://www.exelixis-lab.org/web/software/pear), and then barcodes were filtered and trimmed. The “Gold” database containing the ChimeraSlayer reference database in the Broad Microbiome Utilities^33^ (http://microbiomeutil.sourceforge.net/) was used with UCHIME software^34^ for chimera detection and removal. The remaining reads were clustered into operational taxonomic units (OTUs) using a closed-reference OTU selection protocol at the 97% identity level with a USEARCH algorithm^23^ run against the SILVA database^35^. By using QIIME software^36^, the taxonomy associated with each OTU was assigned as the taxonomy associated with the reference sequence defining the OTU.

### Microbial community analyses

To prevent size effects from skewing downstream analysis, samples containing fewer than 1000 high-quality sequences were removed from the analysis. This method resulted in a total of 11,381,154 high-quality sequences from 203 samples over four HCIs that were then used for community-wide analyses. The numbers of reads for each genus whose relative abundance was more than 0.5% were represented by bar charts. We analyzed the sequencing depth accounted for (normalized) prior to the calculation of, for example, observed richness, by using the “vegan” package^37^. To evaluate the amount of diversity contained within communities, alpha diversity analysis was performed through phyloseq R package version 1.19.1^38^ to generate the Observed, Chao1 and Ace richness and Shannon diversity indices. To determine the amount of diversity shared between two communities (beta diversity), Unifrac distances were calculated between all pairs of samples. Unifrac distances were based on the fraction of branch length shared between two communities in a phylogenetic tree^39^. Weighted Unifrac accounts for membership and relative abundance (community structure, considering members and the content of each member together). Principal Coordinate Analysis (PCoA) was applied to summarize Unifrac distance matrices and generate biplots including taxa^40^. The difference in Shannon diversity indices between each HCI was determined by the ANOVA test and Scheffe post hoc test. Student t-tests were used to test whether the relative abundance of known hospital-associated pathogens differed significantly among the four HCIs. The core microbiota in the environments included genera with >0.5% abundance on average in each HCI. Venn diagrams were obtained by using the bioinformatics & Evolutionary Genomics software^41^. SourceTracker was applied, treating the human-associated gut and oral communities and floor communities as sources and the environment in each HCI as sinks^42^. Community typing was employed using the DMM model supplied in Dirichlet Multinomial R package version 1.16.0^43^. The analysis was performed to confirm that we had obtained the minimum Laplace approximation used as the criteria for selecting the number of community types^16^. Samples assigned to their community types were visualized based on the maximum posterior probability. Genera frequencies in the HCI clusters were generated, and sample distribution across community types was compared.

## Electronic supplementary material

Supplementary information

Supplementary information

## References

[1] Tringe SG (2008). The airborne metagenome in an indoor urban environment. PLoS One..

[2] Chen CH (2016). Dynamic change of surface microbiota with different environmental cleaning methods between two wards in a hospital. Appl Microbiol Biotechnol..

[3] March A (2010). Colonization of residents and staff of a long-term-care facility and adjacent acute-care hospital geriatric unit by multiresistant bacteria. Clin Microbiol Infect..

[4] Chen KH, Chen LR, Wang YK (2014). Contamination of medical charts: an important source of potential infection in hospitals. PLoS One..

[5] Hewitt KM (2013). Bacterial diversity in two Neonatal Intensive Care Units (NICUs). PLoS One..

[6] Tang CY (2015). Application of 16S rRNA metagenomics to analyze bacterial communities at a respiratory care centre in Taiwan. Appl Microbiol Biotechnol..

[7] Oberauner L (2013). The ignored diversity: complex bacterial communities in intensive care units revealed by 16S pyrosequencing. Sci Rep..

[8] Tringe SG, Hugenholtz P (2008). A renaissance for the pioneering 16S rRNA gene. Curr Opin Microbiol..

[9] Martiny JB (2006). Microbial biogeography: putting microorganisms on the map. Nat Rev Microbiol..

[10] Rintala H, Pitkaranta M, Toivola M, Paulin L, Nevalainen A (2008). Diversity and seasonal dynamics of bacterial community in indoor environment. BMC Microbiol..

[11] Tang JW (2009). The effect of environmental parameters on the survival of airborne infectious agents. J R Soc Interface..

[12] Kembel, S. W. *et al*. Architectural design influences the diversity and structure of the built environment microbiome. *ISME J*. **6**, 1469–1479; doi:ismej2011211 (2012). 10.1038/ismej.2011.211 PMC3400407 22278670

[13] Ijaz MK, Zargar B, Wright KE, Rubino JR, Sattar SA (2016). Generic aspects of the airborne spread of human pathogens indoors and emerging air decontamination technologies. American Journal of Infection Control..

[14] Sattar SA (2016). Decontamination of indoor air to reduce the risk of airborne infections: studies on survival and inactivation of airborne pathogens using an aerobiology chamber. American Journal of Infection Control..

[15] Li Y (2007). Role of ventilation in airborne transmission of infectious agents in the built environment - a multidisciplinary systematic review. Indoor Air..

[16] Holmes I, Harris K, Quince C (2012). Dirichlet multinomial mixtures: generative models for microbial metagenomics. PLoS One..

[17] Fierer N, Hamady M, Lauber CL, Knight R (2008). The influence of sex, handedness, and washing on the diversity of hand surface bacteria. Proc Natl Acad Sci USA.

[18] Flores GE (2011). Microbial biogeography of public restroom surfaces. PLoS One..

[19] Wood M (2015). Athletic equipment microbiota are shaped by interactions with human skin. Microbiome..

[20] Lax S (2014). Longitudinal analysis of microbial interaction between humans and the indoor environment. Science..

[21] Meadow JF (2014). Bacterial communities on classroom surfaces vary with human contact. Microbiome..

[22] Ferrari B, Winsley T, Ji M, Neilan B (2014). Insights into the distribution and abundance of the ubiquitous candidatus Saccharibacteria phylum following tag pyrosequencing. Sci Rep..

[23] Edgar RC (2010). Search and clustering orders of magnitude faster than BLAST. Bioinformatics..

[24] Grice EA, Segre JA (2011). The skin microbiome. Nat Rev Microbiol..

[25] Klevens RM (2007). Estimating health care-associated infections and deaths in U.S. hospitals, 2002. Public Health Rep..

[26] Pramono AK, Sakamoto M, Iino T, Hongoh Y, Ohkuma M (2015). Dysgonomonas termitidis sp. nov., isolated from the gut of the subterranean termite Reticulitermes speratus. Int J Syst Evol Microbiol..

[27] Wiener-Well Y (2011). Nursing and physician attire as possible source of nosocomial infections. Am J Infect Control..

[28] Núñez S, Moreno A, Green K, Villar J (2000). The stethoscope in the Emergency Department: a vector of infection?. Epidemiol Infect..

[29] Heyba M (2015). Microbiological contamination of mobile phones of clinicians in intensive care units and neonatal care units in public hospitals in Kuwait. BMC Infect Dis..

[30] Bures S, Fishbain JT, Uyehara CF, Parker JM, Berg BW (2000). Computer keyboards and faucet handles as reservoirs of nosocomial pathogens in the intensive care unit. Am J Infect Control..

[31] Cai L, Ye L, Tong AH, Lok S, Zhang T (2013). Biased diversity metrics revealed by bacterial 16S pyrotags derived from different primer sets. PLoS One..

[32] Zhang J, Kobert K, Flouri T, Stamatakis A (2014). PEAR: a fast and accurate Illumina Paired-End reAd mergeR. Bioinformatics..

[33] Haas BJ (2011). Chimeric 16S rRNA sequence formation and detection in Sanger and 454-pyrosequenced PCR amplicons. Genome Res..

[34] Edgar RC, Haas BJ, Clemente JC, Quince C, Knight R (2011). UCHIME improves sensitivity and speed of chimera detection. Bioinformatics..

[35] Quast C (2013). The SILVA ribosomal RNA gene database project: improved data processing and web-based tools. Nucleic Acids Res..

[36] Caporaso JG (2010). QIIME allows analysis of high-throughput community sequencing data. Nat Methods..

[37] Oksanen J (2013). H. vegan: Community Ecology Package. R package version.

[38] McMurdie PJ, Holmes S (2013). “phyloseq: An R package for reproducible interactive analysis and graphics of microbiome census data”. PLoS ONE..

[39] Lozupone C, Knight R (2005). UniFrac: a new phylogenetic method for comparing microbial communities. Appl Environ Microbiol..

[40] Huang YT, Hsueh PR (2008). Antimicrobial drug resistance in Taiwan. Int J Antimicrob Agents..

[41] Shade A, Handelsman J (2012). Beyond the Venn diagram: the hunt for a core microbiome. Environ Microbiol..

[42] Knights D (2011). Bayesian community-wide culture-independent microbial source tracking. Nat Methods..

[43] Morgan, M. DirichletMultinomial: Dirichlet-Multinomial Mixture Model Machine Learning for Microbiome Data. R package version 1.16.0 (2016).

