# Supplementary material for: Bacterial diversity among four healthcare-associated institutes in Taiwan
Source: Sci Rep. 2017 Aug 15;7:8230. doi: 10.1038/s41598-017-08679-3 (PMC5557925; doi:10.1038/s41598-017-08679-3)
Supplement: Supplementary file 1 — Supplementary information [file 41598_2017_8679_MOESM1_ESM.pdf]

Title: Bacterial diversity among four healthcare-associated institutes in Taiwan

Authors: Chang-Hua Chen, Yaw-Ling Lin, Kuan-Hsueh Chen, Wen-Pei Chen, Zhao-Feng Chen, Han-Yueh Kuo, Hsueh-Fen Hung, Chuan Yi Tang, Ming-Li Liou

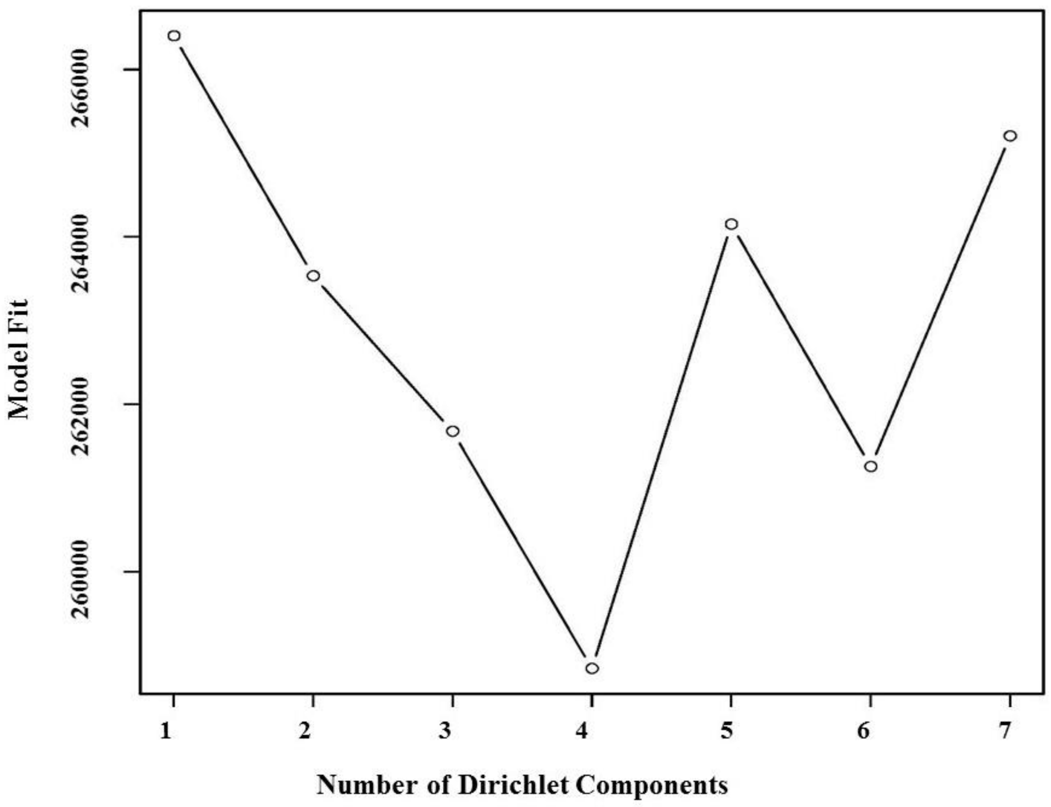

Figure S1.

Fitting the OTUs data from 203 environmental samples to DMM provided support four types when using the Laplace approximation to the negative log model evidence.
