# Supplementary material for: Bacterial diversity among four healthcare-associated institutes in Taiwan
Source: Sci Rep. 2017 Aug 15;7:8230. doi: 10.1038/s41598-017-08679-3 (PMC5557925; doi:10.1038/s41598-017-08679-3)
Supplement: Supplementary file 2 — Supplementary information [file 41598_2017_8679_MOESM2_ESM.doc]

Title: Bacterial diversity among four healthcare-associated institutes in Taiwan

Authors: Chang-Hua Chen, Yaw-Ling Lin, Kuan-Hsueh Chen, Wen-Pei Chen, Zhao-Feng Chen, Han-Yueh Kuo, Hsueh-Fen Hung, Chuan Yi Tang, Ming-Li Liou

| Table S1 | | | | |
| --- | --- | --- | --- | --- |
| Description of healthcare-associated institute samples collected. | | | | |
|  | TH | SC | NH | NC |
| Workplaces | 47 | 6 | 10 | 11 |
| High-touch areas | 48 | 16 | 15 | 13 |
| Environments | 21 | 13 | 3 | 0 |
| Total | 116 | 35 | 28 | 24 |

Notes:

1 Sampling sites around a bed in each health-care institutes were chosen based on the frequency with which the surfaces were highly touched, and the sampling sites were divided into three groups: (1) workplaces, including keyboards, computer mice, curtains, mattresses, and quilts; (2) high touch areas, including beds, monitors, ventilators, stethoscopes, oxygen supply, suction buttons, hemodialysis machines, intravenous pumps and feeding pumps; and (3) environments, including floors.

2 The selection of Sampling sites is modified from Sehulste et al’s report ( Sehulster L, Chinn RY, CDC, HICPAC. Guidelines for environmental infection control in health-care facilities. Recommendations of CDC and the Healthcare Infection Control Practices Advisory Committee (HICPAC). MMWR Recomm Rep. 2003;52(RR-10):1-42.)

Table S2. The Comparison of genus abundance between different institutes and locations.

| Genus / Locations | TH | | | |  | SC | | | |  | NH | | | |  | NC | | | |
| --- | --- | --- | --- | --- | --- | --- | --- | --- | --- | --- | --- | --- | --- | --- | --- | --- | --- | --- | --- |
|  | *Mean* | *SD* | *t/F* | *p* |  | *Mean* | *SD* | *t/F* | *p* |  | *Mean* | *SD* | *t/F* | *p* |  | *Mean* | *SD* | *t/F* | *p* |
| *Propionibacterium* |  |  | 17.09 | .000^***^ |  |  |  | 1.91 | .164 |  |  |  | 1.37 | .273 |  |  |  | .82 | .424 |
| ➀Workplaces | .19 | .15 | ➀ > ➁, ➂ | |  | .01 | .01 |  |  |  | .02 | .01 |  |  |  | .05 | .09 |  |  |
| ➁High-Touch Areas | .06 | .06 |  |  |  | .01 | .01 |  |  |  | .01 | .01 |  |  |  | .03 | .02 |  |  |
| ➂Environments | .08 | .09 |  |  |  | .02 | .02 |  |  |  | .01 | .00 |  |  |  | -- | -- |  |  |
| *Acinetobacter* |  |  | 2.78 | .07 |  |  |  | 1.59 | .220 |  |  |  | 1.06 | .362 |  |  |  | .10 | .923 |
| ➀Workplaces | .10 | .13 |  |  |  | .08 | .12 |  |  |  | .03 | .05 |  |  |  | .03 | .04 |  |  |
| ➁High-Touch Areas | .16 | .17 |  |  |  | .04 | .04 |  |  |  | .09 | .15 |  |  |  | .03 | .02 |  |  |
| ➂Environments | .09 | .11 |  |  |  | .15 | .24 |  |  |  | .01 | .00 |  |  |  | -- | -- |  |  |
| *Dysgonomonas* |  |  | 8.92 | .000^***^ |  |  |  | .78 | .467 |  |  |  | .43 | .655 |  |  |  | 1.00 | .328 |
| ➀Workplaces | .17 | .15 | ➂ > ➀, ➁ | |  | .01 | .01 |  |  |  | .50 | .19 |  |  |  | .38 | .28 |  |  |
| ➁High-Touch Areas | .13 | .14 |  |  |  | .01 | .02 |  |  |  | .53 | .23 |  |  |  | .27 | .28 |  |  |
| ➂Environments | .30 | .20 |  |  |  | .02 | .02 |  |  |  | .63 | .22 |  |  |  | -- | - |  |  |
| *Streptococcus* |  |  | 2.91 | .059 |  |  |  | .78 | .468 |  |  |  | 1.92 | .168 |  |  |  | -.93 | .365 |
| ➀Workplaces | .04 | .05 |  |  |  | .15 | .22 |  |  |  | .01 | .02 |  |  |  | .02 | .04 |  |  |
| ➁High-Touch Areas | .02 | .01 |  |  |  | .10 | .20 |  |  |  | .00 | .00 |  |  |  | .04 | .06 |  |  |
| ➂Environments | .03 | .10 |  |  |  | .05 | .06 |  |  |  | .00 | .00 |  |  |  | -- | -- |  |  |
| *Staphylococcus* |  |  | .38 | .688 |  |  |  | 8.59 | .001^**^ |  |  |  | .81 | .457 |  |  |  | -1.68 | .108 |
| ➀Workplaces | .02 | .03 |  |  |  | .07 | .05 | ➁ > ➀, ➂ | |  | .01 | .01 |  |  |  | .02 | .04 |  |  |
| ➁High-Touch Areas | .02 | .02 |  |  |  | .31 | .21 |  |  |  | .01 | .03 |  |  |  | .08 | .11 |  |  |
| ➂Environments | .02 | .02 |  |  |  | .10 | .09 |  |  |  | .00 | .00 |  |  |  |  |  |  |  |

^*^= *p* < 0.05, ^**^ = *p* < 0.01, ^***^ = *p* < 0.001, Statistical testing was based on the ANOVA test or independent-sample t test.

| Table S3. Number of sequences analyzed and observed median of alpha diversity for the 16S rRNA amplicon analyzed. | | | | | | |
| --- | --- | --- | --- | --- | --- | --- |
| Area | Institute | Reads/n | Observed | Chao1 | ACE | Shannon Index |
| Workplaces | TH | 55,178 | 678.00 | 924.66 | 911.55 | 3.51 |
|  | SC | 51,766 | 1001.50 | 1415.49 | 1337.15 | 4.46 |
|  | NH | 28,487 | 323.00 | 440.38 | 417.85 | 2.70 |
|  | NC | 27,919 | 308.00 | 403.47 | 406.45 | 2.71 |
| High-touch areas | TH | 55,362 | 558.50 | 869.05 | 874.57 | 3.49 |
|  | NH | 26,942 | 301.00 | 403.78 | 402.27 | 2.43 |
|  | NC | 45,471 | 495.00 | 788.65 | 750.42 | 3.02 |
|  | SC | 44,818 | 815.00 | 1253.80 | 1182.62 | 3.92 |
| Environments | TH | 93,226 | 954.00 | 1123.26 | 1087.05 | 3.38 |
|  | NH | 16,691 | 276.00 | 361.41 | 350.39 | 2.31 |
|  | NC | - | - | - | - | - |
|  | SC | 42,656 | 835.00 | 1262.61 | 1282.48 | 4.23 |
